# Supplementary material for: The effect of mother-infant group music classes on postnatal depression—A systematic review protocol
Source: PLoS One. 2022 Oct 6;17(10):e0273669. doi: 10.1371/journal.pone.0273669 (PMC9536550; doi:10.1371/journal.pone.0273669)
Supplement: Attachment — Submitted filename: Peer review PLOS one _complete.docx [file pone.0273669.s002.docx]

Review: The effect of mother-infant group music classes on postnatal depression – a systematic review protocol

Altogether, I think this is an interesting research proposal with direct impact on clinical and societal practice. The authors propose a thorough systematic review that follows all relevant guidelines, and I believe that it is a well thought-out setup. I also appreciate the intersectional approach of the proposed analysis, where the authors propose to also look at mothers who are socially disadvantaged.

However, I do think there are some concerns that the authors should address in their study. These are mainly concerned with the inclusion criteria regarding postpartum depression and the risk of selection bias and drop-outs in the studies that are included in the review, which could skew the overall results. I believe that taking these factors into account could improve the quality and generalizability of this study.

- One of the main outcomes of the study is postnatal depression. Could the authors maybe specify which kind of outcomes they will include? Will they only include studies that conducted clinical interviews or clinically validated questionnaires, or will they also include studies that used other questions to measure postpartum depression?
- I see that the inclusion criteria also do not contain a “threshold” or criterium for postpartum depression. Do the authors propose to include all studies that look at effects of music classes on postnatal depression, even when the studies only included women without postpartum depression? Or do they plan to only include studies which included women with postpartum depression, and if so, what are the diagnosis criteria?
  - A short note on the reporting of the participant demographics:
    I think it is also important to report participants’ level of postpartum depression before the onset of the study, so that it is clear to which postpartum depression group the results can be generalized.
- I see that the authors propose to include all studies on group music classes, regardless of the setting in which the class is organized. This might induce a possible “interest bias”, where mothers who are actively seeking out music classes are more likely to participate and benefit from the classes. Do the authors plan on addressing a form of “interest bias”, meaning women who are more interested in the classes might join the classes?
  - This form of interest bias could be accounted for through only including studies which have a control group or “regular care” group as comparison group, but the authors propose to also include studies that have no control group. Do they plan on conducting separate meta-analyses or comparisons for studies with or without a control group?
- My other concern in the included studies is the amount of time and effort it will take the participating mothers to participate in the music classes. These classes could take quite some time, maybe during the day on work days, meaning that maybe mothers with more time on their hands and mothers who feel more motivated and “up for it” might be more likely to complete the study. This could introduce a form of selective drop-out bias, where mothers who have less time or energy might be more likely to drop out.
  - How do the authors plan on addressing drop-out participants per study, and the possible bias this introduces in the results?
- Motherhood and maternity leave are also very dependent on national contexts. The authors do propose to do a subgroup analysis for studies which include socially disadvantaged mothers, but do they plan on accounting for or reporting other international differences, such as national policies on maternity leave or the amount of involvement of the other parent?
  - It could be interesting to report the setting in which participating mothers are in, for example, do the music classes take place in the evenings or during maternity leave so that they do not have to take time off work?

Altogether, I appreciate the rather practical approach of the proposed review. I believe that this study could be an interesting starting point to consider the feasibility of music classes for women with postpartum depression, especially considering the possible improvement of social support and maternal-infant bonding for participating women. I wish the researchers the best of luck with this interesting line of research!
