# Supplementary material for: The effect of mother-infant group music classes on postnatal depression—A systematic review protocol
Source: PLoS One. 2022 Oct 6;17(10):e0273669. doi: 10.1371/journal.pone.0273669 (PMC9536550; doi:10.1371/journal.pone.0273669)
Supplement: Attachment — Submitted filename: Response to Reviewers .docx [file pone.0273669.s003.docx]

| **Reviewer 1 comments** | **Response** |
| --- | --- |
| One of the main outcomes of the study is postnatal depression. Could the authors maybe specify which kind of outcomes they will include? Will they only include studies that conducted clinical interviews or clinically validated questionnaires, or will they also include studies that used other questions to measure postpartum depression? | We will include any measure of postnatal depression. We have now provided further clarity on p10 under the section ‘Primary outcome’ by specifying what kind of outcomes we will include: clinically validated assessment tools, such as the Diagnostic and Statistical Manual of Mental Disorders, fifth edition (DSM-5), as well as self-reported measurement tools. |
| I see that the inclusion criteria also do not contain a “threshold” or criterium for postpartum depression. Do the authors propose to include all studies that look at effects of music classes on postnatal depression, even when the studies only included women without postpartum depression? Or do they plan to only include studies which included women with postpartum depression, and if so, what are the diagnosis criteria? | We will include all studies that look at the effect of music classes on outcomes of interest, such as postnatal depression or maternal anxiety, regardless of inclusion criteria for women. In other words, all women with and/or without symptoms of, for example, postnatal depression or anxiety on recruitment will be included. We have provided clarity for this on p10 under the section ‘Participants’.  We will, however, plan to conduct a subgroup analysis comparing studies with an inclusion criteria of women who were screened and met a given threshold for our primary outcome. Postnatal depression, versus those studies that did not. Further detail on this has now been added to p17 under the section ‘Subgroup analyses’. |
| A short note on the reporting of the participant demographics: I think it is also important to report participants’ level of postpartum depression before the onset of the study, so that it is clear to which postpartum depression group the results can be generalized. | We will report on participants level of postnatal depression at the time of recruitment onto the study, if the study reports baseline scores for this outcome. We have added this detail on p13 when discussing the process for data extraction. |
| I see that the authors propose to include all studies on group music classes, regardless of the setting in which the class is organized. This might induce a possible “interest bias”, where mothers who are actively seeking out music classes are more likely to participate and benefit from the classes. Do the authors plan on addressing a form of “interest bias”, meaning women who are more interested in the classes might join the classes? | We agree with this comment and acknowledge it may be a limitation. A successful randomisation process by randomised controlled trials may reduce the risk of “interest bias” by randomly assigning those individuals, who have consented only because they are actively seeking out music classes, to the intervention or control group; thus, limiting selection bias from this confounder. However, sampling bias may still occur if all those that consent to participate are actively seeking out music classes, meaning that they are not representative of the population of interest. The first of these issues will be addressed under the domain of ‘Selection bias’ within the Cochrane Risk of Bias Tool, while the second may be considered under the ‘Other sources of bias’ domain. For non-randomised controlled studies, it is likely they will be at greater risk of this form of “interest bias”, due to non-randomisation of study participants.  For either type of study, if there is evidence of these sources of bias, it will inform our quality assessment and subsequent discussion on the generalisability of the findings. |
| This form of interest bias could be accounted for through only including studies which have a control group or “regular care” group as comparison group, but the authors propose to also include studies that have no control group. Do they plan on conducting separate meta-analyses or comparisons for studies with or without a control group? | We are only including studies that have a control group. We have enhanced clarity of our intention to exclude any studies without a control group on p11 under the ‘Exclusion criteria’ section. |
| My other concern in the included studies is the amount of time and effort it will take the participating mothers to participate in the music classes. These classes could take quite some time, maybe during the day on work days, meaning that maybe mothers with more time on their hands and mothers who feel more motivated and “up for it” might be more likely to complete the study. This could introduce a form of selective drop-out bias, where mothers who have less time or energy might be more likely to drop out.  How do the authors plan on addressing drop-out participants per study, and the possible bias this introduces in the results? | We agree with this comment and believe it raises two issues of concern: (i) participant drop out from the research study; and, (ii) potential low adherence to the intervention for those randomly assigned to the intervention group and who have less time or energy to attend sessions. We will address each of these issues in the review.  For (i), this will be addressed in the quality assessment of included studies through the Cochrane Risk of Bias Tool within the domain of ‘Attrition bias’. We will make a judgment on the rate of attrition from the study, reasons for attrition (if reported/known), and any imbalance in attrition across groups. We will also assess the impact of any high risk of attrition bias through sensitivity analysis by looking at whether the results are sensitive to dropouts from the research. We have added further detail on this under the section ‘Sensitivity analyses’ on p17.  For (ii), this will be addressed through the ‘Incomplete outcome data’ domain in the Cochrane Risk of Bias Tool, if information is reported by individual studies, to provide an assurance that the principles of intention-to-treat were adhered to for the analysis. As part of the process evaluation, we will also extract information on adherence and reasons for non-adherence, if reported, and discuss accordingly. We have added further detail on p12 |
| Motherhood and maternity leave are also very dependent on national contexts. The authors do propose to do a subgroup analysis for studies which include socially disadvantaged mothers, but do they plan on accounting for or reporting other international differences, such as national policies on maternity leave or the amount of involvement of the other parent? | Thank you for highlighting this interesting consideration. An overview of included studies will be provided in the review including detail on country of origin. It is not planned to conduct a specific sub-group analysis on country of origin however this will be synthesised and discussed narratively with a reflection on the transferability to other settings in light of similarities and differences in national maternity policy approach. We have added additional text under the ‘Discussion’ section on P17. |
| It could be interesting to report the setting in which participating mothers are in, for example, do the music classes take place in the evenings or during maternity leave so that they do not have to take time off work? | We agree that this is an important factor. We will report this when describing the characteristics of included studies. As part of the synthesis of process evaluations, we will look for evidence of this and synthesise narratively. We have clarified this on p12. |

| **Reviewer 2 comments** | **Response** |
| --- | --- |
| The authors provided information for the review in the context of what is already known. However, they wrote that “are not aware of any systematic reviews specifically addressing the effect of mother-infant group music classes on postnatal depression”. It was not clearly stated in the manuscript that a search of resources for existing or ongoing reviews was taken. I recommend to add information what kind of resources/databases have been checked to ensure the current review is justified. | Thank you for identifying this omission. Detail has now been edited on p8 to clarify that searches of Prospero and the Cochrane Library were conducted with no completed or ongoing systematic reviews on this topic identified. |
| I would recommend to consider the piloting the study selection process by applying the inclusion criteria to a sample of papers in order to check that they can be reliably interpreted and that they classify the studies appropriately. | A process of piloting the study selection process was indicated in the manuscript – we have highlighted this text on p13. |
| Please state how extracting data from multiple reports of the same study will be done (Each report separately, then combine information across multiple data collection forms OR data from all reports directly into a single data collection form.) | The Covidence software, which we will use to screen records and extract data from included studies, allows for multiple reports to be merged for the same study at the data extraction stage. We will use this tool to help manage records. Data from multiple reports from the same study will then be extracted using a single data extraction form. We have added further clarity on p13. |
| Please expand all abbreviations used in the manuscript. e.g. EPDS, GAD 7, PHQ-9 was not explained while WHO abbreviation was introduced two times. | This has been completed as requested with the following abbreviations expanded in the manuscript:  On p4: EPDS, GAD-7, PHQ-9  On p10: DASS  On p12: EMBASE, CINAHL, CENTRAL, LILACS  On p.14: ROBINS-I  On p.15: GRADE-CERQual  For the WHO abbreviation, the second expanded text has been removed on p7. |
| I found that a searching strategy is dedicated to RCTs, and since the authors plan to include non-randomized studies as well, it might be wise to not add keywords as “Randomized Controlled Trials” or “Random Allocation”. | Thank you for this comment. The Canada’s Drug and Health Technology Agency (CADTH) search filter will be utilised, which is sensitive to non-randomised controlled trials. This detail has been added for clarification on p12. |
| At the same time, I would be pleased if the authors could consider to restrict the eligibility criteria to RCTs only for the reliability of the data. And if so, my previous comment (no 5) would be not relevant then. | In the meta-analysis, we will prioritise RCT data. If non-randomised controlled trials are found, they will be synthesised separately through a narrative summary. This detail has been added for clarification on p15.  In an area where limited evidence is possible, we want to be as inclusive as possible, and we will be guided by the Cochrane handbook for the quality assessment and handling of data extracted from the included studies. |
